# High MBL-expressing genotypes are associated with deterioration in renal function in type 2 diabetes

**DOI:** 10.3389/fimmu.2022.1080388

**Published:** 2022-12-23

**Authors:** G. H. Dørflinger, P. H. Høyem, E. Laugesen, J. A. Østergaard, K. L. Funck, R. Steffensen, P. L. Poulsen, T. K. Hansen, M. Bjerre

**Affiliations:** ^1^ Medical/Steno Aarhus Research Laboratory, Aarhus University, Aarhus, Denmark; ^2^ Department of Internal Medicine, Regional Hospital Gødstrup, Gødstrup, Denmark; ^3^ Department of Endocrinology and Internal Medicine, Aarhus University Hospital, Aarhus, Denmark; ^4^ Steno Diabetes Center Aarhus, Aarhus University Hospital, Aarhus, Denmark; ^5^ Regional Centre for Blood Transfusion and Clinical Immunology, Aalborg University Hospital, Aalborg, Denmark

**Keywords:** diabetes duration, mannan-binding lectin, MBL genotypes, albuminuria, T2D

## Abstract

**Introduction:**

Accumulating evidence support that mannan-binding lectin (MBL) is a promising prognostic biomarker for risk-stratification of diabetic micro- and macrovascular complications. Serum MBL levels are predominately genetically determined and depend on MBL genotype. However, Type 1 diabetes (T1D) is associated with higher MBL serum levels for a given MBL genotype, but it remains unknown if this is also the case for patients with T2D. In this study, we evaluated the impact of MBL genotypes on renal function trajectories serum MBL levels and compared MBL genotypes in newly diagnosed patients with T2D with age- and sex-matched healthy individuals. Furthermore, we evaluated differences in parameters of insulin resistance within MBL genotypes.

**Methods:**

In a cross-sectional study, we included 100 patients who were recently diagnosed with T2D and 100 age- and sex-matched individuals. We measured serum MBL levels, MBL genotype, standard biochemistry, and DEXA, in all participants. A 5-year clinical follow-up study was conducted, followed by 12-year data on follow-up biochemistry and clinical status for the progression to micro- or macroalbuminuria for the patients with T2D.

**Results:**

We found similar serum MBL levels and distribution of MBL genotypes between T2D patients and healthy individuals. The serum MBL level for a given MBL genotype did not differ between the groups neither at study entry nor at 5-year follow-up. We found that plasma creatinine increased more rapidly in patients with T2D with the high MBL expression genotype than with the medium/low MBL expression genotype over the 12-year follow-up period (p = 0.029). Serum MBL levels did not correlate with diabetes duration nor with HbA1c. Interestingly, serum MBL was inversely correlated with body fat percentage in individuals with high MBL expression genotypes both at study entry (p=0.0005) and 5-years follow-up (p=0.002).

**Discussion:**

Contrary to T1D, T2D is not *per se* associated with increased MBL serum level for a given MBL genotype or with diabetes duration. Serum MBL was inversely correlated with body fat percentage, and T2D patients with the high MBL expression genotype presented with deterioration of renal function.

## Introduction

Increasing evidence supports that activation of the complement system plays an important role in developing diabetes-related micro- and macrovascular complications (1, 2). Mannan-binding lectin (MBL) is a serum protein primarily produced in the liver with the ability to distinguish between self and non-self-cells based on carbohydrate pattern recognition. MBL-binding to carbohydrate patterns initiates the lectin pathway, which leads to inflammatory cell recruitment, opsonization, and membrane attack complex formation (3). Hyperglycemia is associated with altered carbohydrate patterns present on the self-cell surface (4). Thus, MBL may cause inexpedient complement activation and tissue injury through binding to glycated self-tissue.

Serum MBL levels are largely determined genetically by six common single nucleotide polymorphisms (SNPs) in the *MBL2* gene, resulting in large inter-individual serum levels of MBL (5–7). The six SNPs in the *MBL2* gene are divided into three MBL expression genotypes; low MBL expression (O/O) with serum MBL levels below (≤100 µg/L), medium MBL expression (A/O) with serum MBL levels (101–1,000 µg/L), and high expression MBL (A/A) with serum MBL (>1,000 µg/L) (5, 8).

We and others have shown that high expression MBL genotypes and high MBL serum levels are associated with an increased risk of both micro- and macrovascular complications in patients with diabetes (9–12). Additionally, we observed a significantly increased mortality in patients with T1D and the high MBL expression genotype as compared with the patients with a “non-high-expression” MBL genotype (13). High MBL level has been reported as an independent marker of diabetic nephropathy and cardiovascular disease both in patients with T1D (9, 14) and T2D (15, 16). To our knowledge, no longitudinal studies have been performed in regard to MBL levels and development in renal function.

Several studies have found associations between serum MBL levels and insulin resistance (17, 18), though in nondiabetic settings low serum MBL levels were associated with insulin resistance. A study of weight loss and changes in insulin resistance and serum MBL levels showed that weight loss-induced changes in serum MBL concentration were positively associated with the increase in insulin sensitivity (18). This indicates that MBL levels are influenced by the degree of insulin resistance.

We have previously found significantly higher serum MBL levels among patients with T1D compared to healthy subjects (9, 12). However, the distribution of MBL genotypes did not differ between the groups (12) and therefore did not explain the phenotypic differences. These findings are supported by significantly higher serum MBL in patients with new-onset juvenile T1D as compared with their non-diabetic siblings matched for high-expression MBL genotype (19) as well as in animal studies (20).

It remains unknown if T2D *per se* is associated with altered MBL serum levels for a given MBL genotype. We have previously found comparable serum MBL levels in T2D and healthy individuals, but the relation to MBL genotype distribution was not clarified (21).

The current study aimed to investigate MBL serum level and MBL genotype in newly diagnosed T2D patients compared to age- and sex-matched healthy individuals. Additionally, we examined the relationship between MBL and renal decline as well as diabetes duration and insulin resistance.

## Methods

### Participants

100 patients with type 2 diabetes (T2D) were consecutively recruited from the outpatient clinic and 100 healthy subjects (controls) matched for age and sex were included in the study as previously described (22). Inclusion criteria at study entry were >18 years of age and, for patients, <5 years duration since diagnosis of diabetes. The diabetes-related treatment for all patients with type 2 diabetes was managed by their general practitioner according to the normal guidelines including insulin-treatment, other antihyperglycemic treatments than insulin, and non-pharmacological treatment such as guidance in a lifestyle intervention. The healthy subjects were recruited by advertising in the local press, and excluded if diabetes was diagnosed by fasting glucose and oral glucose tolerance tests. General exclusion criteria were: acute or chronic infectious disease, end-stage renal failure, pregnancy or lactation, prior or present cancer, and contraindications to MRI scanning (claustrophobia, magnetic material in the body, and body weight > 120 kg). Three T2D patients and one healthy individual were excluded from the study due to missing genotype, no serum MBL sample, or withdrawal of consent.

The participants were invited for a 5-year follow-up visit and 63 patients with T2D and 72 healthy controls attended (23). In total, 37 T2D patients dropped out during follow-up for the following reasons: follow-up invitation rejected (n=24), no contact (n=6), GAD-positive (n=2), deaths (n=5). Twenty-eight participants from the control group dropped out during follow-up for the following reasons: follow-up invitation rejected (n=22), no contact (n=2), death (n=4). For this study, we obtained a dual-energy x-ray absorptiometry (DEXA), blood, and urine sampling, and the medical history of the participants was obtained by a questionnaire at both visits. A 12-year follow-up on clinical diabetes status was also performed: HbA1c, UACR, creatinine, and CRP were obtained from the medical records for the patients with T2D (n = 54).

The study was conducted according to the Declaration of Helsinki and was approved by the local Ethical Committee (1-10-72-349-13) and by the Danish Data Protection Agency (1-16-02-505-13), Denmark. All participants gave their written, informed consent to participate.

### Blood analyses

Fasting blood samples were obtained from the antecubital vein. The serum was separated and stored at -80°C until further analysis. Serum MBL levels were measured using an in-house time-resolved immune-fluorometric assay with a lower detection level of 10 µg/L, as described previously by (24). The intra- and inter-assay variations (%CV) were below 10%. High-sensitive C-reactive protein (hsCRP) levels were quantified by an in-house assay as previously described by (25). The limit of detection was 0.005 µg/L. The intra- and inter-assay variations (%CV) were below 5 and 6%, respectively.

All other blood and urine samples were analyzed with accredited methods at the Department of Clinical Biochemistry at Aarhus University Hospital.

### MBL expression genotypes

Genomic DNA was extracted from whole blood using the Maxwell 16 System Blood DNA Purification Kit (Promega, Madison, WI, USA) according to the manufacturer’s protocol. Genotyping for six SNPs (single-nucleotide polymorphism) in the *MBL2* gene was performed using a real-time polymerase chain reaction with TaqMan SNP Genotyping Assays (Applied Biosystems, Foster City, CA, USA) as previously described by (26). Three SNPs are located within the promoter region of the *MBL2 gene* (rs11003125, rs7096206, rs12780112)]) and three SNPs (rs1800450, rs5030737, rs1800451) are located in exon 1 of the *MBL2 gene.* Because of linkage disequilibrium, the six SNPs give rise to seven major haplotypes: HYPA, LYQA, LYPA, LXPA, LYPB, LYQC, and HYPD that were further categorized into three MBL expression genotypes; low (O/O), medium (A/O), and high (A/A) as previously described (5, 7). These MBL expression genotypes has previously been shown to be correlated with serum MBL levels below (≤100 µg/L), (101–1,000 µg/L), and (>1,000 µg/L), respectively (8).

### Blood pressure

Ambulatory blood pressure (BP) was measured at 20-min intervals for 24 h using Spacelab 90217 (Spacelabs Healthcare, Issaquah, Washington, USA) in between study days. Office BP was measured on the right arm with an appropriately sized cuff, and mean SBP and DBP were calculated as the average of three measurements obtained by an oscillometric BP monitor (Riester Champion N; Riester GmbH; Jungingen, Germany) after more than 5 min of rest in the seated position. Mean arterial BP (MAP) was calculated as DBP + 0.4 × pulse pressure (PP).

### Body composition

A dual-energy radiograph absorptiometry scan (DEXA Discovery System; Hologic, Marlborough, Massachusetts, USA) was performed to estimate lean and fat body mass.

### Urinary albumin-to-creatine ratio and estimated glomerular filtration rate

Urinary albumin excretion (UAE) was evaluated by albumin-to-creatinine ratios (ACR) in three morning urine samples. Patients were classified as microalbuminuric when at least two of three samples had urinary albumin-to-creatine ratios of 2.5:25 mg/mmol (men) and 3.5:35 mg/mmol (women) or above. The estimated glomerular filtration rate (eGRF) was calculated from the MDRD study equation from serum creatinine (27).

### Statistics

Data are presented as mean ( ± SD) if normally distributed and median (IQR) if non-normally distributed. Given the exploratory aims of the cohort study, power calculations were not carried out. MBL measurements below assay detection limit was set to 10 µg/L. Comparisons between groups were performed using an unpaired t-test for normally distributed data and Mann-Whitney’s U-test for non-normally distributed data. Comparisons of paired data points were achieved with the Wilcoxon signed Rank sum test. Multiple linear regression (Generalized Linear Model) was used to allow adjustment for confounders. The Chi-square test tested the difference in distribution between non-continuous variables. Spearman’s correlation analysis was used to estimate the strength of the association between non-normally distributed variables. P-values were considered significant if <0.05. To characterize the effect of MBL expression genotype on different biomarkers mixed model ANOVA was performed, where data passed the normality test. All calculations were performed in R.

## Results

Clinical characteristics of the T2D patients and their age- and sex-matched healthy individuals are shown in **Table 1**. The diabetes duration at study entry was 1.9 years (IQR: 0.7;3.2). All the participants had well-adjusted treatment, regarding glycemia, blood lipids, and blood pressure. The T2D patients had a higher body fat percentage, HbA1c, albumin/creatinine ratio (ACR), and hsCRP as compared to the healthy individuals. A significantly higher proportion of the T2D group was treated with statins, which may explain their significantly lower total- and LDL- cholesterol levels compared to the control group. Similarly, the T2D group was more often treated with one or more anti-hypertensive medications, which was probably the reason for T2D patients not having higher blood pressure than the control group. Data from 63 patients with T2D and 72 controls were available for 5-year follow-up (**Table 2**) and the baseline characteristics for the paired group have previously been described (28).

**Table 1:** Characteristics of the participants at study entry. Data are mean ± SD unless other indicated.

	T2D Patients (n=100)	Healthy controls (n=100)	P-value
Sex (male/female) (%)	52/48	52/48	
Age (years)	58 ( ± 9.8)	58 ( ± 9.7)	0.892
Diabetes duration at study entry (years) (median (IQR))	1.9 (2.4)	–	–
Body Mass Index (kg/m^2^)	30 ( ± 4.7)	26 ( ± 4.0)	<0.001
Fat percent (%)	32.8 ( ± 8.4)	28.8 ( ± 8.2)	0.001
HbA1c (mmol/mol)	48.0 ( ± 7.0)	37.9 ±( ± 3.8)	<0.001
Total-cholesterol (mmol/L)	4.4 ( ± 0.8)	5.7 ( ± 1.0)	<0.001
LDL-cholesterol (mmol/L)	2.3 ( ± 0.7)	3.4 ( ± 1.0)	<0.001
HDL-cholesterol (mmol/L)	1.4 ( ± 0.3)	1.7 ( ± 0.6)	<0.001
Triglycerides (mmol/L)	1.6 ( ± 0.7)	1.4 ( ± 0.7)	0.036
Urine-albumin/creatinine ratio (mg/mmol) (median (IQR))	2.76 (4.23)	2.01 (2.13)	0.001
Estimated glomerular filtration rate (eGFR) (ml/min/1.73m²) (median (IQR))	87.4 (22.5)	81.6 (16.2)	0.01
High sensitive CRP (mg/L) (median (IQR))	1.83 (2.68)	0.79 (1.01)	<0.001
24-h ABPM systolic BP (mmHg)	126 ( ± 18.8)	125 ( ± 12.5)	0.625
24-h ABPM diastolic BP (mmHg)	74 ( ± 7.4)	76 ( ± 7.7)	0.186
Smoking (present/previous/never) (%)	21/36/42	21/33/46	0.732
Diabetes treatment (insulin or insulin+other antihyperglycemic/other antihyperglycemic other/lifestyle intervention) (%)	8/65/27	–	–
Statin treatment (%)	76	18	<0.001
Antihypertensive treatment (n %)	63	25	<0.001
Mannan binding Lectin (MBL) (median (IQR))			
Total group (µg/L) Low MBL expression genotype (O/O), (µg/L) Medium MBL expression genotype (A/O),(µg/L) High MBL expression genotype (A/A), (µg/L)	643 (1152.5) 15 (15.5), (n=15) 421 (412), (n=41) 1522 (1259), (n=42)	798 (1394) 24 (30), (n=13) 402 (557.5), (n=43) 1691 (1255), (n=43)	0.274 0.527 0.564 0.384

**Table 2:** Characteristics of the participants at 5-year follow-up. Data are mean ± SD unless other indicated.

	T2D Patients (n=63)	Healthy controls (n=72)	P-value
Sex (male/female) (n)	33/30	36/36	0.784
Age (years)	65.4 ( ± 9.6)	63.2 ( ± 10)	0.200
Diabetes duration at study entry (years) (median (IQR))	7.7 (2.5)		
Body Mass Index (kg/m^2^)	30.2 ( ± 5.5)	26.8 ( ± 4.1)	<0.001
Fat percent (%)	34.7 ( ± 8.3)	31.2 ( ± 8.0)	0.016
HbA1c (%)	52.8 ( ± 11.6)	38.2 ( ± 3.4)	<0.001
Total-cholesterol (mmol/L)	3.98 ( ± 1.03)	5.3 ( ± 1.1)	<0.001
LDL-cholesterol (mmol/L)	1.9 ( ± 0.84)	3.1 ( ± 1.02)	<0.001
HDL-cholesterol (mmol/L)	1.3 ( ± 0.41)	1.6 ( ± 0.6)	<0.001
Triglycerides (mmol/L)	1.84 ( ± 1.8)	1.13 ( ± 0.9)	0.006
Urine-albumin/creatinine ratio (mg/mmol) (median (IQR))	3.3 ( ± 8)	0.6 ( ± 3.3)	0.012
High sensitive CRP (mg/L) (median (IQR))	1.8 (2.7)	1.1 (2.35)	0.39
24-h ABPM systolic BP (mmHg)	127.8 ( ± 13.5)	129.3 ( ± 12.4)	0.49
24-h ABPM diastolic BP (mmHg)	74.4 ( ± 7.3)	78.9 ( ± 7.8)	0.001
Smoking (present/previous/never) (%)	27/9/27	22/12/38	0.161
Diabetes treatment (insulin or insulin+other antihyperglycemic/other antihyperglycemic/lifestyle intervention) (%)	11/45/44		
Statin treatment (n)	53	16	<0.001
Antihypertensive treatment (n)	48	26	0.001
Mannan binding Lectin (MBL) (median (IQR))			
Total group (µg/L) Low MBL expression genotype (O/O), (µg/L) Medium MBL expression genotype (A/O), (µg/L) High MBL expression genotype (A/A), (µg/L)	337 (1154) 10 (0.50), (n=11) 295 (205), (n=27) 1548 (1012), (n=24)	584 (1215) 16 (18), (n=10) 316 (231), (n=27) 1585 (1273), (n=32)	0.108 0.025 0.426 0.682

**Figure 1:**
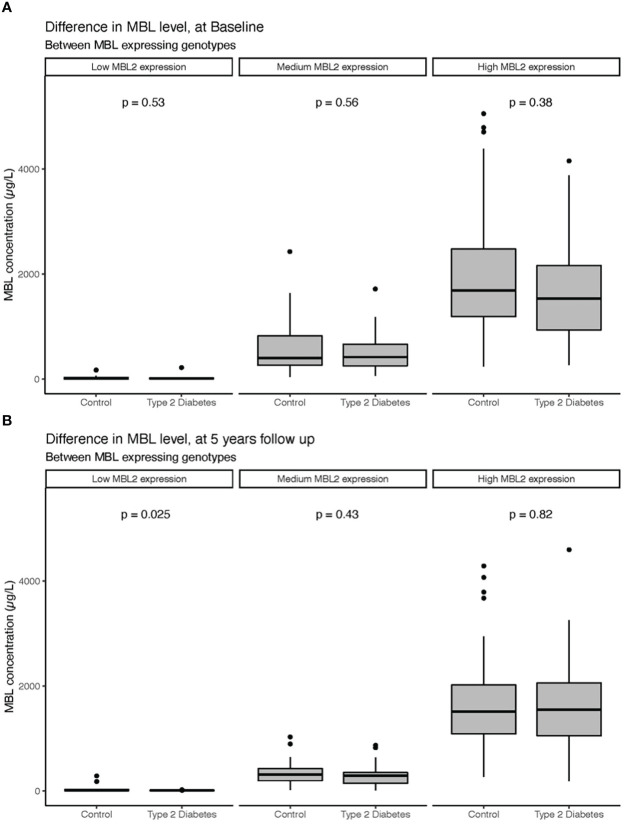
Serum levels of MBL at study entry **(A)** and 5-year follow-up **(B)** in patients with T2D and healthy controls divided according to MBL expressing genotypes. Bars represent medians, and boxes indicate IQRs. P-values are calculated with the Mann-Whitney U-test for differences between groups.

Distribution of MBL genotype (low-, medium- and high -expression) in the T2D group was 14,4%, 42,3% and 43,3%, respectively, which was similar to the control group, 13%, 43% and 43% (p=0.78). Serum MBL levels (regardless of genotype) did not differ between the T2D group and the control group (643 μg/L (IQR 251;1404) vs. 798 μg/L (IQR 266.5;1660.5), respectively, (p=0.274)) at study entry (**Figure 1A**) or after 5-years follow up (**Figure 1B**). Serum MBL levels remained similar in the two groups after adjustment for HbA1c, urine-albumin/creatinine ratio, antihypertensive treatment, and statin treatment in a multiple linear regression analysis.

### Change in MBL level over time:

**Figure 2:**
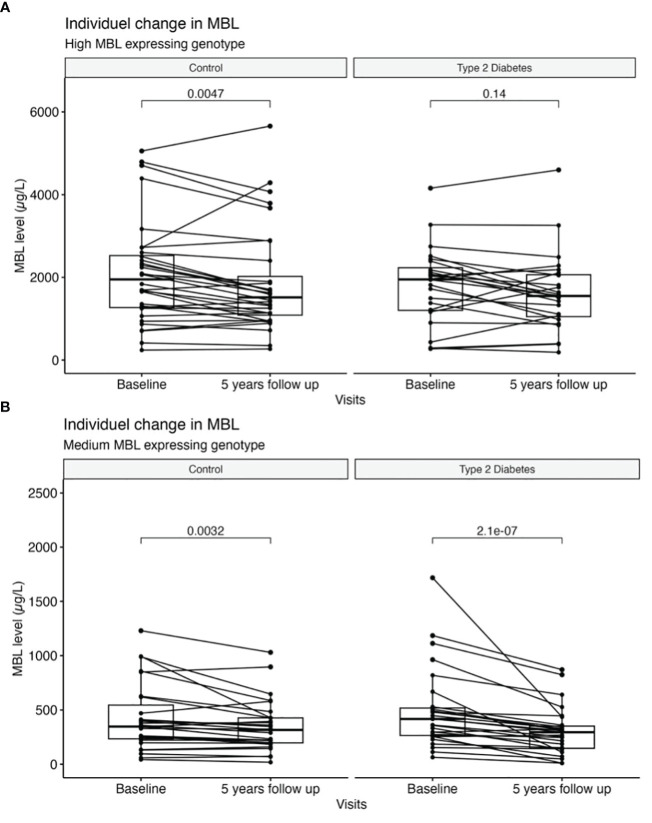
Individual change in serum levels of MBL between baseline and 5-year follow-up. **(A)** High MBL expressing genotypes and **(B)** medium MBL expressing genotypes are divided according to diabetes status. Bars represent medians, and boxes indicate IQRs. P-values are calculated with the Wilcoxon signed-rank test for differences between groups.

MBL levels in paired samples, for T2D patients with high MBL expression genotype, was 1949 μg/L (IQR 1200;2231) at study entry and 1548 μg/L (1051;2063) at the 5-year follow-up visit (p= 0.143) (**Figure 2A**). Analyzing the MBL ratio in a multiple linear regression model diabetes status (p= 0.01) and a high MBL expressing genotype (p= 0.005) seems to influence the outcome. MBL levels decreased significantly in the high MBL expression healthy control group over the 5 years from 1950 μg/L (IQR 1168;2526) at study entry and to 1514 μg/L (IQR 1087;2022) after 5 years of follow-up, p=0.005 (**Figure 2A**). A similar decrease in serum MBL levels was found in the medium MBL expressing genotypes, where MBL levels decreased over the 5-year follow-up from 417 μg/L (IQR 265;518) to 295 μg/L (IQR: 147;352) for patients with T2D (p<0.001) and 347 μg/L (IQR 234;545) to 316 (IQR 197;427) in the healthy control subjects (p=0.003) (**Figure 2B**).

MBL was reduced in the low MBL expression genotype of patients with T2D, 15 μg/L (IQR 11;21) at study entry versus 10 μg/L (IQR 10;11) p=0.025 and in the control group, 24 μg/L (IQR: 10;30) to 16 μg/L (IQR: 11;29) p=0.4. However, due to the low sample size and values near the assay detection limit, the statistic should be interpreted cautiously in this subgroup. Adjustments for HbA1c, ACR, antihypertensive treatment, and statin treatment respectively did not change the results, neither in the total cohort nor after subdividing by MBL expressing genotype.

### MBL difference between sex:

Men had higher serum MBL levels compared to women in the entire study group (991 μg/L (IQR 299;1831) vs. 460 μg/L (IQR 186; 1024), p= 0.004. This was also the case with men in the control group who had significantly higher serum MBL levels compared to women (1107 μg/L (IQR 318; 1950) vs. 442 μg/L (IQR 207; 1148), p= 0.03). However, the serum MBL levels was barely significantly higher in men with T2D compared to women (820 μg/L (IQR 296;1767) vs. 478 μg/L (IQR 192;933), p= 0.054). Unequal distribution of MBL genotypes between men and women in either group did not explain the difference.

### Correlations between MBL, body fat %, and insulin resistance:

**Figure 3:**
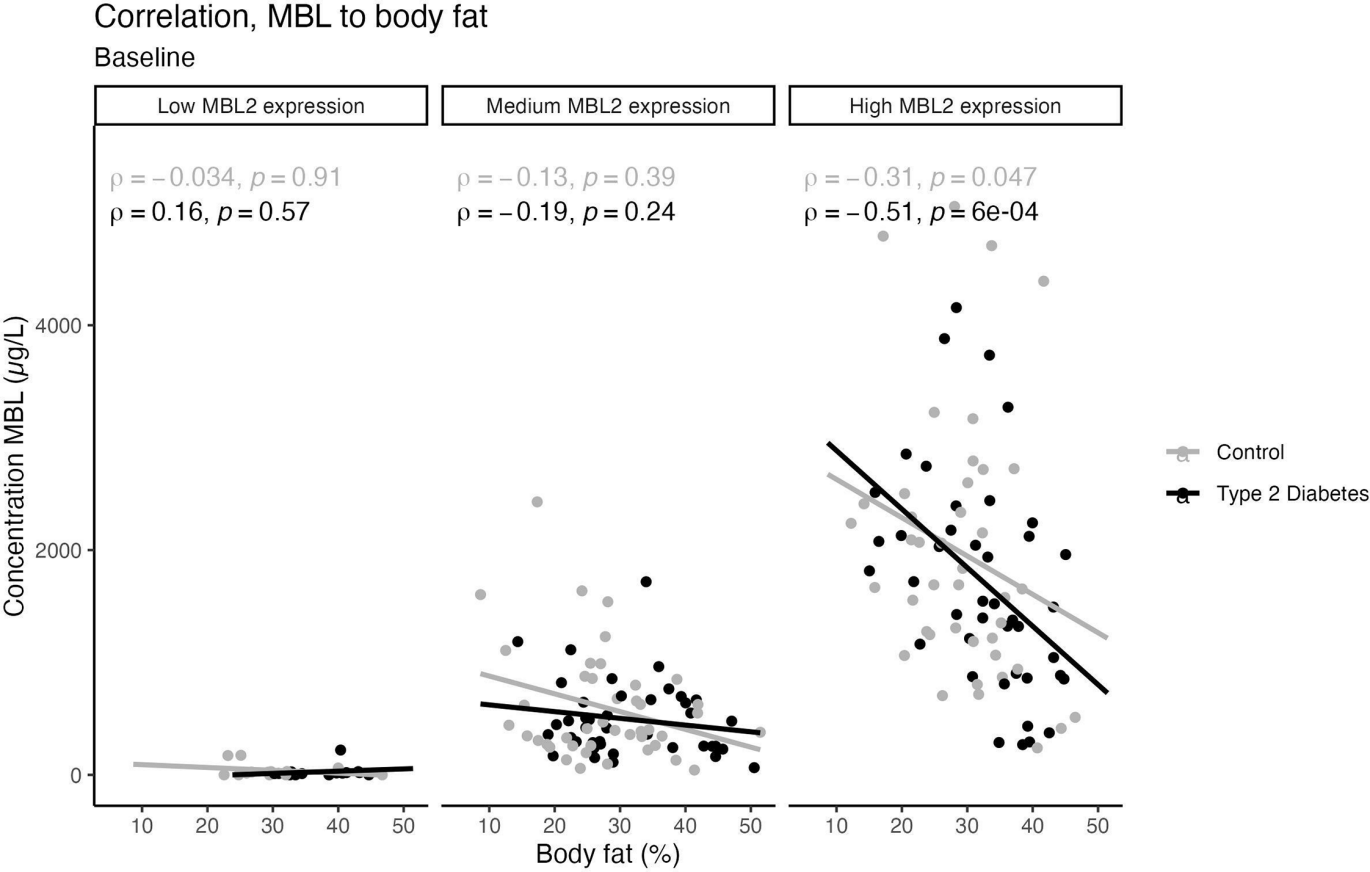
Scatter plots with trend lines showing a possible linear relationship between body fat percentage and Serum MBL levels, divided by MBL expressing genotype. Grey dots represent healthy individuals and the black dots represent participants with T2D. Spearman’s correlation analysis was used to generate the correlation coefficients and associated p-value.

A weak inverse correlation between serum MBL level and body fat percentage was present in the entire group (ρ=-0.21, p=0.003). The outcome was not influenced by either HbA1c, antihypertensive treatment, statin treatment, or diabetes status when analyzing data in a multiple linear regression analysis. However, MBL expressing genotype significantly influenced the correlation between MBL and body fat percentage (p< 0.001). Subdividing by MBL genotype, a negative correlation with fat percentage was present for the high MBL expression genotype (ρ= -0.51, p=0.0005) (**Figure 3**). No correlations were found in medium or low MBL expression genotypes, which might be due to the low sample size. At the 5-year follow-up, these correlations were still present in the total cohort (ρ= -0.268, p=0.002).

No correlations were found between MBL and HOMA-β or HOMA-IR and no significant differences in MBL serum levels were found in T2D patients treated with insulin compared to those treated with other anti-diabetic medication or with lifestyle intervention alone. Also, no significant correlations were found between serum MBL level and HbA1c, hsCRP, total-, LDL- or HDL-cholesterol respectively at either study entry or 5-year follow-up (**Table 3**). Statin treatment or any antihypertensive treatment was not significantly associated with altered MBL serum levels.

**Table 3:** An overview of Spearman’s correlation analysis (r) and p-value for serum MBL levels and different variables according to diabetes status.

	MBL			
	T2D		Healthy controls	
Variables	ρ	p-value	ρ	p-value
Diabetes duration (years)	0.106	0.299	–	–
HbA1c (mmol/mol)	-0.181	0.075	0.071	0.487
UACR (mg/mmol)	-0.052	0.612	0.001	0.992
eGFR (ml/min/1.73m²)	-0.184	0.070	-0.19	0.061
CRP (mg/L)	-0.133	0.191	0.051	0.615
BMI (kg/m^2^)	-0.358	<0.001	-0.049	0.633
Fat percent	-0.271	0.007	-0.130	0.201

### The difference in biomarkers for nephropathy 12-year follow-up:

**Figure 4:**
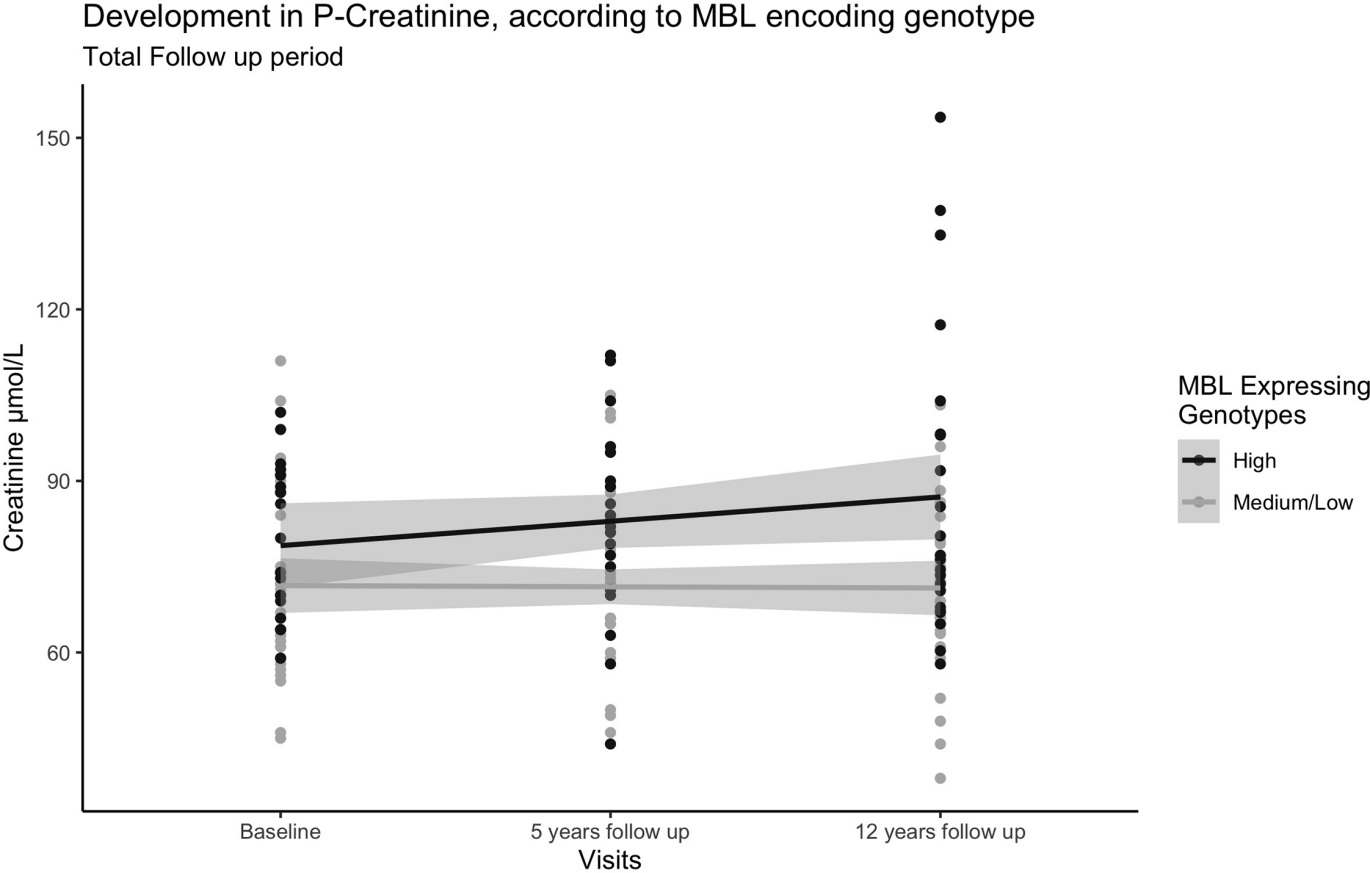
Dot plot with trend lines showing development in plasma creatinine in participants with T2D over the 12-year follow-up period. Black points represent participants with the high MBL expressing genotype (n=22) and the grey points represent participants with medium/low MBL expression genotypes (n=30). A mixed model ANOVA was performed to compare the effect of the MBL genotype on the development of plasma creatinine.

In participants with T2D, both eGFR and plasma creatinine differed significantly between high and medium/low expressing genotypes after 12 years of follow-up. The median for eGFR was 70.0 ml/min/1.73m² (IQR 55.5;85.5) in the high MBL expression genotype and 76.9 ml/min/1.73m² (IQR 80.9; 90.0) in the medium/low MBL expression genotype, (p= 0.012). Plasma creatinine was significantly higher in the high MBL expression genotype (76.7 µmol/L (IQR 68.6;98.2)) as compared to 70.0 µmol/L (IQR 68.6;98.2) in the medium/low MBL expression genotype (p= 0.02). A mixed model ANOVA was performed to compare the effect of the MBL genotype on the development of plasma creatinine. For patients with T2D, we found that plasma creatinine increased more rapidly in the high MBL expression genotype than in the medium/low MBL expression genotype (**Figure 4**), F(2, 100) = 4.09, p = 0.029. Statistical testing of hsCRP, HbA1c and the ACR did not uncover any significant differences after 12-years of follow-up.

Data from 54 patients with were available for our final analysis (Baseline characteristics of participants attending versus those not attending the follow-up visit are listed in **Table S1**). Participants lost to follow-up were significantly younger (p= 0.036) and had lower levels of low-density lipoprotein cholesterol (p= 0.012).

## Discussion

We showed a similar distribution of MBL genotypes in newly diagnosed T2D patients as in age- and sex-matched healthy individuals. The distribution of MBL expression genotype in healthy individuals has previously been reported in a large Danish population study (n=9245) (29). They showed an MBL expression genotype frequency of 58% for high (A/A), 37% for the middle (A/O), and 5% for low (or MBL deficient O/O). We have recently shown a frequency slightly shifted towards MBL deficiency (A/A 54.5%, A/O 30.8%, and O/O 14.7%) in a group (n=3043) of newly diagnosed T2D patients (16). This distribution is similar to the distribution found in the present study.

Here we showed that serum MBL levels for a given MBL expression genotype were not altered in patients with recently diagnosed T2D as opposed to what has previously been observed in patients with T1D. In a group of patients with T2D and suspected acute myocardial infarction (30), the distribution of MBL genotypes was comparable to the background population, however, as a group, the T2D patients with AMI had higher serum MBL levels than previously reported in the background population of T2D patients, which may be related to vascular stress (30). Although serum MBL levels are known to be determined largely by polymorphisms in the *MBL2* gene, differences in circulating MBL levels of up to 10-fold can be found between individuals despite identical genotypes (6).

Portal hypoinsulinemia is present when the portal system is bypassed in subcutaneous insulin administration, as opposed to the pancreas-secreted insulin. The increased serum MBL level for a given genotype seen in T1D patients (9) has generated the hypothesis that the portal hypoinsulinemia characteristic of T1D may increase hepatic MBL synthesis. T2D, on the other hand, is characterized by insulin resistance, including hepatic insulin resistance. Thus, it seems plausible that T2D patients may also have high MBL serum levels for a given MBL genotype because of hepatic insulin resistance. However, the current study did not find evidence of this mechanism. A possible explanation for the lack of stimulation could be that hepatic insulin resistance is counteracted by increased endogenous insulin secretion, and as a net result does not affect MBL levels in T2D or the fact that the patients with T2D were not significantly more insulin resistant than the control group. The inclusion criteria included a maximum diabetes duration of 5 years. *In vitro* studies with human hepatocytes supported clinical observations of hormonal influence on MBL synthesis, with a significant increase in MBL expression after incubation with thyroid hormones and growth hormone (31), whereas insulin and IGF-1 had no effect. Interestingly, we found that MBL levels decreased after 5-years of follow-up, in healthy individuals with the high MBL expression genotype and remained unchanged in patients with T2D and was not explained by weight gain or change in fat percent.

We found a highly significant inverse correlation between MBL and fat percentage, determined by a whole-body DEXA scan, a very precise expression for obesity. This finding is supported by other studies. In women with polycystic ovary syndrome, obesity was associated with lower MBL levels (17). Among non-diabetic men, MBL serum level was significantly lower in obese subjects than in lean subjects. Further, MBL was significantly correlated with insulin sensitivity as assessed by euglycemic-hyperinsulinemic clamp (18). In a study of nine morbidly obese women, MBL levels also correlated with insulin sensitivity (32). An alternative explanation for the inverse associations between MBL levels and insulin sensitivity, suggests that MBL acts as an anti-inflammatory protein by promoting phagocytic clearance of various inflammatory agents, which would in turn cause subjects with low MBL levels to develop chronic low-grade inflammation, and ensuing insulin resistance or obesity. However, we have shown that MBL levels are unaffected by significant weight loss in obese subjects (33). In the current study, we found no correlation between MBL and hsCRP, an accepted marker of low-grade inflammation. We, therefore, find the hypothesis of MBL as an anti-inflammatory protein less plausible and inclined to the view that MBL secretion is under the regulation of insulin.

It seems somehow contradictive though, that fat percent (followed by insulin resistance) is inversely correlated with MBL levels, whereas T2D patients (insulin resistant) overall have similar MBL levels to age- and sex-matched healthy individuals. We speculate that in obese, non-diabetic individuals, insulin resistance is overcome by increased endogenous insulin secretion, making their net insulin action in the liver able to suppress MBL production. At the time when endogenous insulin secretion can no longer counteract insulin resistance (at the debut of T2D) the net insulin action in the liver is lower and does not overall suppress MBL, although, within the group of T2D, there is still an increasing suppression of MBL with increasing fat percent and obesity.

We found that MBL serum levels were inversely associated with fat percent and triglycerides in the group of T2D patients at study entry and also after 5 years of diabetes duration for the fat percent. A common pathway in the form of peroxisome proliferator-activated receptor-α (PPARα) for regulating MBL levels and triglycerides has been suggested by (34). PPARs are ligand-activated transcription factors known to regulate glucose, fatty acid and lipoprotein metabolism, energy balance, and inflammation, among others (35). Along with the regulation of lipid and glucose metabolism, PPARα is an attractive candidate gene for the risk of metabolic syndrome and T2D. Hepatic *MBL2* gene expression and circulating MBL levels are reported to be stimulated by PPARα and fenofibrate in humans, linking PPAR to the regulation of innate immunity and complement activation in humans thus suggesting a possible role of MBL in lipid metabolism (34).

The present study shows an increasing plasma creatinine concentration over the 12-years follow-up period in the T2D patients with the high MBL expression genotype as compared to the medium/low MBL expression genotype. This corresponds with our previous findings, that the high MBL expression genotype was more frequent in T1D patients with diabetic nephropathy than in those with normal urinary albumin excretion (10) and that the presence of high MBL genotypes was associated with a 1.5-fold increased risk of developing nephropathy compared to patients with a ‘low expression’ MBL genotype (9) in patients with T1D. Also, serum MBL concentrations were significantly higher in patients with macroalbuminuria as compared to patients with normal albumin excretion rate, which persisted in the group of patients with high MBL expression genotypes (10), however, no SNPs in the *MBL2* gene were reported to confer risk of T1D or diabetic nephropathy. Cai et al. showed that serum and urine MBL levels were higher in patients with T2D and diabetic nephropathy who were prone to develop end-stage renal failure (36), and several studies have reported an association between high plasma MBL levels and the development of diabetic nephropathy in patients with T2D (15, 37, 38). In contrast, Adrian et al. showed that the MBL genotype was not associated with long-term clinical effects in patients with end-stage renal disease, however, only 8% of the patients in the study had diabetes (39). We have recently shown a U-shaped association between serum MBL, high MBL expression genotypes, and risk of cardiovascular events in a large group of patients newly diagnosed with T2D but not with all-cause mortality, supporting the role of MBL in vascular complications (16).

## Limitations

The group of T2D patients is newly diagnosed, and well-regulated and may therefore not be representative of T2D patients in general. Unfortunately, only 63% (T2D) and 72% (healthy controls) of the individuals were able or willing to participate in the 5-years follow-up study and we were only able to receive 12-year follow-up biochemistry from patients with T2D but no data from the healthy control group.

## Conclusion

In conclusion, we found similar MBL serum levels for any given MBL genotype between T2D patients and matched healthy control subjects. Furthermore, we found a significant inverse correlation between fat percentage and serum MBL levels in the high MBL expression genotype, but the mechanism for this finding needs to be further investigated. We show deterioration of renal function, illustrated by lower eGFR and increased serum creatinine in T2D patients with the high MBL expression genotype.

## Data availability statement

The original contributions presented in the study are included in the article/**Supplementary Material**. Further inquiries can be directed to the corresponding authors.

## Ethics statement

The studies involving human participants were reviewed and approved by the Central Denmark Region Committees on Health Research Ethics (1-10-72-349-13) and by the Danish Data Protection Agency (1-16-02-505-13), Denmark. The patients/participants provided their written informed consent to participate in this study.

## Author contributions

Conception and study design: GD, JØ, TH and MB. Patient recruitment and collection of clinical samples: PH, EL, KF, PP and TH. Methods and data analysis: GD, RS and MB. Manuscript drafts: GD and MB. All authors interpreted the data. All authors contributed to the article and approved the submitted version.

## Supplementary material

Supplementary Table 1 Baseline characteristics in participants included and not included in the 12 years follow-up data-analysis
