# Supplementary material for: High MBL-expressing genotypes are associated with deterioration in renal function in type 2 diabetes
Source: Front Immunol. 2022 Dec 23;13:1080388. doi: 10.3389/fimmu.2022.1080388 (PMC9816478; doi:10.3389/fimmu.2022.1080388)
Supplement: Supplementary Table 1 — Baseline characteristics in participants included and not included in the 12 years follow-up data-analysis [file Table_1.docx]

Supplementary table S1: Baseline characteristics of participants included and not included in the 12-year follow-up data analysis.

|  | Type 2 Diabetes | | P-value |
| --- | --- | --- | --- |
|  | 12 years follow-up  (n= 54) | Dropout  (n= 44) |  |
| Sex (male/female) (%) | 30/24 | 21/23 | 0.45 |
| Age (years) | 60.4 (±9.2) | 56.2 (±10.3) | 0.036 |
| Diabetes duration at study entry (years) (median (IQR) | 2.1(2.1) | 1.6 (2.6) | 0.18 |
| Body Mass Index (kg/m^2^) | 29.8 (±4.8) | 30.3 (±4.9) | 0.57 |
| Fat percent (%) | 31.6 (±8.5) | 34.1 (±8.2) | 0.15 |
| HbA1c (mmol/mol) | 47.9(±7.4) | 48.0 (±6.7) | 0.90 |
| Total-cholesterol (mmol/L) | 4.3(±0.8) | 4.5(±0.9) | 0.18 |
| LDL-cholesterol (mmol/L) | 2.1 (±0.7) | 2.5 (±0.8) | 0.012 |
| HDL-cholesterol (mmol/L) (median (IQR) | 1.4 (0.48) | 1.3 (0.43) | 0.33 |
| Triglycerides (mmol/L) (median (IQR) | 1.4 (1) | 1.3 (0.7) | 0.22 |
| Urine-albumin/creatinine ratio (mg/mmol) (median (IQR)) | 0.5 (0.7) | 0.4 (0.4) | 0.19 |
| Estimated glomerular filtration rate (eGFR) (ml/min/1.73m²) (median (IQR)) | 81.2 (21.5) | 93.1 (24.3) | 0.03 |
| High sensitive CRP (mg/L) (median (IQR) | 1.4 (1.7) | 2.2 (3.3) | 0.04 |
| 24-h ABPM systolic BP (mmHg) (median (IQR) | 125 (13) | 123 (13) | 0.63 |
| 24-h ABPM diastolic BP (mmHg) (median (IQR) | 74 (8) | 74 (8) | 0.63 |
| Smoking (present/previous/never) (%) | 9/20/25 | 11/16/16 | 0.26 |
| Diabetes treatment (insulin or insulin+other antihyperglycemic/other antihyperglycemic/ lifestyle intervention) (%) | 6/31/17 | 0/32/10 | 0.12 |
| Statin treatment (%) | 27.9 | 23.3 | 0.40 |
| Antihypertensive treatment (n %) | 33.3 | 39.5 | 0.53 |
| Mannan binding Lectin (MBL) (median (IQR))  Total group (µg/L)  Low MBL expression genotype (O/O), (µg/L)  Medium MBL expression genotype (A/O),(µg/L)  High MBL expression genotype (A/A), (µg/L) | 440 (1465)  17 (21.5)  417(218)  1949 (1163) | 734 (1062)  11.5 (53)  518 (423)  1411 (1241) | 0.39  0.95  0.86  0.52 |
